# Combined toxic effects of aflatoxin B_2_ and the protective role of resveratrol in Swiss albino mice

**DOI:** 10.1038/s41598-021-95879-7

**Published:** 2021-09-10

**Authors:** Alperen Gündüz, Emine Yalçın, Kültiğin Çavuşoğlu

**Affiliations:** Department of Biology, Faculty of Science and Art, Giresun University, 28200 Giresun, Turkey

**Keywords:** Biochemistry, Biotechnology, Molecular biology

## Abstract

In this study, the toxic effects of aflatoxin B_2_ (AFB_2_) on Swiss albino mice and the protective effects of resveratrol were investigated. Physiological (body weight, liver and kidney weight), biochemical (aspartate aminotransferase-AST, alanine transaminase-ALT, blood urea nitrogen-BUN, creatinine, malondialdehyde-MDA and glutathione-GSH) and cytogenetic parameters (micronucleus-MN in buccal epithelium, erythrocyte and leukocyte cells and chromosomal aberrations-CAs) were used to determine the toxic effects. Additionally, scavenging effects of resveratrol against superoxide, hydrogen peroxide (H_2_O_2_) and 1,1-diphenyl-2-picrylhydrazyl (DPPH) radicals were also investigated. In experimental period, mice were divided into six groups and the groups were treated with tap water, 10 mg/kg b.w resveratrol, 20 mg/kg b.w resveratrol, 20 µg/kg b.w. AFB_2_, 10 mg/kg b.w resveratrol + 20 µg/kg b.w AFB_2_, 20 mg/kg b.w resveratrol + 20 µg/kg b.w AFB_2_, respectively. As a result, the scavenging effects of resveratrol increased with increasing dose and the superoxide, H_2_O_2_ and DPPH radical scavenging activity of resveratrol were 74.9%, 79.1% and 49.2%, respectively. AFB_2_ administration caused a significant decrease in physiological parameters, and these decreases regressed in AFB_2_ + resveratrol treated groups. Serum ALT and AST activities, BUN and creatinine levels were higher in the AFB_2_ treated group compared to the control group and serious abnormalities were found in MDA and GSH levels in the kidney and liver. In the group treated with AFB_2_ + 20 mg/kg resveratrol, ALT, AST, BUN and creatinine levels decreased significantly and GSH levels increased compared to only-AFB_2_ treated group. AFB_2_ triggered MN formation in buccal epithelium, erythrocyte and leukocyte cells and CAs in bone marrow cells. The application of 20 mg/kg resveratrol together with AFB_2_ was decreased the MN and CAs frequency. Resveratrol exhibited a recovery effect in the range of 40.9–80.5% against AFB_2_ toxicity in all tested parameters. In this study, it was determined that AFB_2_ caused serious changes in selected physiological, biochemical and cytogenetic parameters while resveratrol displayed a protective role against these toxic effects.

## Introduction

Mycotoxins are secondary metabolites produced by fungi in a wide variety of agricultural products depending on ecological conditions. These toxins are produced by some molds such as *Penicillium*, *Aspergillus* and *Fusarium* under certain temperature and humidity conditions and cause deterioration in agricultural products. Until today, more than 400 different types of mycotoxins have been identified, and fumonisin, ochratoxin, aflatoxin, zeranol, trichothecenes and patulin are common and well-known mycotoxin types. Aflatoxins are a type of mycotoxin that threaten many agricultural products and cause toxic effects on human health^1,2^. Due to the high humidity and temperature in feeds, grains and dry foods, *Aspergillus* species proliferate rapidly and produce aflatoxin. Aflatoxins produced by *Aspergillus flavus*, *Aspergillus nomius* and *Aspergillus parasiticus* are classified into six groups as B_1_, B_2_, G_1_, G_2_, M_1_ and M_2_ according to their fluorescence properties and their movements in the chromatogram^3^. Exposure to aflatoxins occurs in different ways. Exposure through direct ingestion of aflatoxin contaminated food, consumption of products such as milk and cheese from animals fed with contaminated feed, or inhalation of aflatoxin dust particles in contaminated food industries and factories are some of these routes. Aflatoxins are highly fat-soluble compounds and readily pass into the bloodstream from the site of exposure, usually through the gastrointestinal tract and respiratory tract^4–6^. Long-term consumption of aflatoxin-containing foods causes serious health problems.

It is known that aflatoxins have mutagenic, carcinogenic and teratogenic effects and cause acute and chronic toxicity especially in kidney and liver. Aflatoxins listed as aflatoxin B_1_ > aflatoxin G_1_ > aflatoxin B_2_ > aflatoxin G_2_ according to the strength of their toxic effects. The best defined and researched aflatoxin type until today is B_1_^1,7^. In this study, the toxic effects of aflatoxin B_2_ (AFB_2_), about which we have less information among the aflatoxin species, were investigated. AFB_2_, produced by *A. flavus*, *A. arachidicola*, *A. nomius*, *A. minisclerotigenes* and *A. parasiticus*, is a dihydro derivative of AFB_1_^8,9^. AFB_2_ is metabolized in the liver by microsomal monooxygenases to the less toxic reactive metabolite AFM_2_. It is also possible for AFB_2_ to be metabolized to AFB_1_ in some cases^10,11^. Although there are not many studies on the toxic effects of AFB_2_ on human health, it has dangerous hepatotoxic, teratogenic and carcinogenic effects on various farm animals^8,12^.

Human exposure to a large number and variety of xenobiotics causes many adverse health effects and various diseases. In order to reduce all these negative effects and to protect against cancer, diabetes and heart diseases, it is necessary to consume natural active ingredients regularly in the daily life. Epidemiological studies have revealed that people can be protected against many diseases by regular consumption of natural and active compounds. Especially many diseases associated with oxidative stress can be prevented by consuming compounds with antioxidant activity^13,14^. Oxidative stress and endogenous antioxidant defense system in the body are in a balance, and disruption of this balance causes oxidative damage. Therefore, in addition to endogenous antioxidants, dietary exogenous antioxidants also contribute to the restoration of this balance. Resveratrol, which is intensively synthesized in legumes, pine trees and especially grapes, is a phytoalexin with antioxidant property. Resveratrol exhibits a protective activity by directly scavenging free radicals, regulating the expression and activity of antioxidant enzymes such as glutathione (GSH) peroxidase, superoxide dismutase (SOD) and catalase (CAT)^15,16^. In addition, resveratrol prevents induced lipid peroxidation and low density lipoprotein (LDL) oxidation and has an effective scavenging effect against hydroxyl, superoxide and metal-radicals. It is also reported that it prevents DNA damage induced by free radicals and disruption in cell membrane integrity^17^. Resveratrol shows anti-oxidative, anti-inflammatory activity and prevents cytotoxic effects by activating mechanisms such as mitochondrial biogenesis and autophagy. Resveratrol is also protective against many diseases such as diabetes, neurodegenerative disorders, cognitive disorders, cancer, kidney diseases and cardiovascular diseases^18–21^.

In this study, the potential toxic effects of AFB_2_ on albino mice and the combined protective effects of resveratrol against these toxic effects were investigated by using physiological, biochemical and cytogenetic parameters. Physiological effects were determined by body weight gain, liver and kidney weight analyzes, and the biochemical effects were determined by serum parameter analyzes and oxidant/antioxidant dynamic. For cytogenetic effects, chromosomal aberrations (CAs) in bone marrow cells and micronucleus (MN) frequency in erythrocyte, leukocyte and buccal mucosa epithelium cells were investigated. And also superoxide, hydrogen peroxide (H_2_O_2_) and 1,1-diphenyl-2-picrylhydrazyl (DPPH) scavenging effects of resveratrol were studied.

## Materials and methods

### Materials

AFB_2_ and the other chemicals were supplied from Sigma-Aldrich. Resveratrol (Solgar Vitamin and Herb, Leonia, NJ, USA) was obtained commercially from local markets.

### Scavenging activity of resveratrol

In this study, free radical scavenging activity of resveratrol was tested against superoxide, DPPH and H_2_O_2_.

### Superoxide radical scavenging activity

The superoxide radical scavenging activity of resveratrol was studied according to the modified method proposed by Gülçin^22^. Light induction of the reaction mixture was carried out using a fluorescent lamp (20 W). A mixture (3 mL) of riboflavin (1.33 × 10^−5^ M), methionine (4.46 × 10^−5^ M) and nitroblue tetrazolium (8.15 × 10^−8^ M) was prepared and illuminated at 25 °C for 40 min. The un-illuminated mixture was used as a blank. Different concentrations of resveratrol (0.125–1.0 mg/mL) were added to the reaction medium and the absorbance of all mixtures were measured at 560 nm. The reduced absorption of the reaction mixture indicates increased scavenging activity. The percentage of superoxide scavenging activity was calculated using Eq. (1).

1 $$\text{Superoxide} \; \text{scavenging} (\%) = [1-({\text{A}}_{1}/{\text{A}}_{2})] \times 100$$

where A_1_ is the absorbance of resveratrol or standard solution, A_2_ is the absorbance of the control. The experiment was repeated three times at each concentration.

### Hydrogen peroxide scavenging activity

H_2_O_2_ scavenging experiments were carried out according to the procedure of Ruch et al.^23^. A solution containing different concentrations of resveratrol (3.4 mL, 0.125–1.0 mg/mL) and 0.6 mL H_2_O_2_ (40 mM) was prepared. The absorbance of the reaction mixture was measured spectrophotometrically at 230 nm. Sodium phosphate buffer, which does not contain H_2_O_2_, accepted as blank. The scavenging activity was determined by monitoring the decrease in H_2_O_2_ absorbance. The absorbance of H_2_O_2_ and the scavenging activity of resveratrol were calculated using Eqs. (2) and (3), respectively.

2 $$\mathrm{Absorbance} \; \left({\lambda }_{230}\right)= 0.505 \times \left[{\text{H}}_{2}{\text{O}}_{2}\right]$$

3 $${\text{H}}_{2}{\text{O}}_{2} \; \text{scavenging} \; \text{activity} (\%) = [1-({\text{A}}_{1}/{\text{A}}_{2})] \times 100$$

A_1_ is the absorbance of solution in the presence of resveratrol, A_2_ is the absorbance of the control. The experiment was repeated three times at each concentration.

### DPPH radical scavenging assay

DPPH scavenging activity of resveratrol was determined using the method suggested by Blois^24^. This method is based on the principle of de-coloring DPPH in methanol solution. DPPH creates a purple color in the methanol solution and turns yellow in the presence of antioxidant substances. 2.4 mL DPPH (0.1 mM) solution in methanol was mixed with different concentrations of 1.6 mL resveratrol (0.125–1.0 mg/mL). The absorbance of the solution left in the dark for 30 min was measured spectrophotometrically at 517 nm. BHT was used as standard and DPPH radical scavenging activity was calculated using Eq. (4).

4 $$\% \; \text{DPPH} \; \text{radical} \; \text{scavenging} \; \text{activity} = ({\text{A}}_{0}- {\text{A}}_{1})/{\text{A}}_{0}\times 100$$

A_0_ is the absorbance of the control and A_1_ is the absorbance of the resveratrol or standard solution. The experiment was repeated three times at each concentration.

### Experimetal design

36 male *Mus musculus* var. *albinos* were used as subjects. During the experiment, 36 albino mice were maintained on a 12 h light/12 h dark cycle, in steel stainless (26 × 15 × 50) at a temperature of 22 ± 3 °C and a relative humidity of 55 ± 5%. All experiments were performed in accordance with the guidelines of the Animal Experiments Local Ethics Committee of Giresun University and approved by the Animal Ethics Committee of Giresun University (protocol number: 2017/02). This study was carried out in compliance with the ARRIVE guidelines.

In order to determine the effects of AFB_2_ and resveratrol applications, thirty-six albino mice were randomly divided into 6 groups with 6 mice per each group. As shown in the Table 1, the group fed only drinking water and standard pellet feed was accepted as the control group and coded as group I. 10 mg/kg and 20 mg/kg b.w of resveratrol were administered and these groups were coded as Group II and III, respectively. The toxic effects of AFB_2_ alone were determined in 20 µg/kg b.w AFB_2_ applied group coded as Group IV. For determining the dose-dependent protective effects of resveratrol against potential toxic effects of AFB_2_, different doses of resveratrol and AFB_2_ were administered together, and these groups were coded as Group V and Group VI. Groups and group formation principle are given in Table 1. The water, feed and solutions of the groups were checked daily during 28 days of application. At the end of the administration period, all mice were sacrificed. The doses of AFB_2_ and resveratrol application were determined according to the doses indicated in literature studies and where clear effects were observed^25,26^.

**Table 1:** Groups and grouping principles.

Groups	Treatment
Group I	Tap water and standard pellet feed
Group II	10 mg/kg b.w resveratrol
Group III	20 mg/kg b.w resveratrol
Group IV	20 µg/kg b.w. AFB_2_
Group V	10 mg/kg b.w resveratrol + 20 µg/kg b.w AFB_2_
Group VI	20 mg/kg b.w resveratrol + 20 µg/kg b.w AFB_2_

### Body weight, liver and kidney weights

Before the application and at the end of the 28-day application period, the weights of the albino mice belonging to each group were measured with the help of sensitive scale after being anesthetized. At the end of the 28th day, after the sacrificing of each mouse, liver and kidney organs were taken and their weights were measured with a sensitive scale.

### Liver and kidney functions analysis

At the end of the administration period, blood samples were collected by cardiac puncture with the animal under mild ether anesthesia. For serum isolation blood samples centrifuged at 1.200 rpm for 10 min. AST (Catalog Number: A559-150) and ALT (Catalog Number: A524-150) enzyme activities, BUN (Catalog Number: B549-150) and creatinine (Catalog Number: C513-480) levels were measured by using Teco Diagnostic kits^27^.

### Antioxidant/oxidant dynamics

In the experimental groups each mice was sacrificed under Halothane anesthesia by heart exaggeration method and then liver and kidneys were removed, washed and homogenized for biochemical analysis. Homogenization was performed in a cold 0.15 M KCl bath at 16.000 rpm with a homogenizer (Ultraturrax Type T25-B, IKA Labortechnik). Homogenates were centrifuged at 5000 rpm at 4 °C for 1 h, and malondialdehyde (MDA) and glutathione (GSH) analyzes were performed in supernatants^28^. Tissue MDA and GSH levels were determined according to the colorimetric method suggested by Yoshoiko et al.^29^ and by Beutler^30^, respectively.

### Cytogenetic analysis

MN and CAs frequency were used for cytogenetic analysis. In order to determine the cytogenetic effects of AFB_2_ and resveratrol applications, the frequencies of MN in buccal mucosa epithelial cells, erythrocytes and leukocytes were determined. In order to determine MN frequency, the criteria suggested by Fenech et al.^31^ were applied. 1.000 cells were counted in each group in order to detect the frequency of MN in all three cell types.

### MN frequency in buccal mucosa epithelial cells

In order to detect the frequency of MN in the buccal mucosa epithelial cells, the mouths of anesthetized mice were washed with distilled water, and samples were taken from the right and left cheek mucosa epithelial cells. Cells were placed on sterile slides and fixed with a 3:1 methanol/acetic acid solution. The slides were stained with Fast Green and examined with an IM-450 TI model research microscope^32^.

### MN frequency in erythrocytes

MN analysis applied on erythrocytes was performed according to the method suggested by Te-Hsiu et al.^33^. Blood samples taken from tail veins of anesthetized mice with the help of injector were subjected to MN analysis. 5 µL of blood sample was mixed with 3% EDTA solution, spread on sterile slides and fixed in 70% ethanol. The slides stained with 5% Giemsa for 15 min and examined under IM-450 TI model research microscope to determine the frequency of MN in erythrocytes.

### MN frequency in leukocyte

In order to determine the frequency of MN in leukocyte cells, blood samples taken from mice were centrifuged at 5000 rpm for 10 min. 0.075 M KCl solution was added to the pellet and kept at room temperature for 20 min. At the end of the period, the tubes were centrifuged at 5000 rpm for 10 min, the pellet was incubated with methanol:glacial acetic acid (3:1) solution. At the end of the incubation, leukocyte cells were spread on sterile slides, stained with 5% Giemsa and examined under IM-450 TI model research microscope^34^.

### CAs frequency in bone marrow

Another test used to determine cytogenetic effects is the CAs test and bone marrow cells were used to identify CAs. For this aim, 0.025% colchicine was administered interperitoneally to the mice in the experimental groups before sacrification and bone marrow was aspirated from the femurs of the sacrified mice at the end of the experimental stages. Cells treated with KCl (0.075 M) were fixed with Carnoy's solution. The fixed cells were stained with Giemsa (5%) and CAs were classified according to the criteria suggested by Savage^35^.

### Recovery effect of resveratrol

The protective effects of resveratrol (Recovery effect = RE) against AFB_2_-induced toxicity were calculated using Eq. (5). Data from Group VI, in which resveratrol provided the highest protection, and Group IV, in which AFB_2_ was administered alone, and data from the control group were used to determine RE. RE values were calculated separately for all tested parameters.

5 $$\text{RE} (\%) = [({\text{D}}_{1}-{\text{D}}_{2}) /({\text{D}}_{3}-{\text{D}}_{2})] \times 100$$

D_1_ is the data of AFB_2_ + resveratrol treated group, D_2_ is the Data of AFB_2_ treated group, D_3_ is the data of control.

### Statistical analysis

Statistical analysis of the findings obtained as a result of the experimental procedures was performed using the SPSS for Windows V 22.0 (SPSS Inc, Chicago, IL) program, and Duncan and One-way ANOVA tests were used to evaluate the statistically significant differences between the experimental groups. Results are given as mean ± SEM and were considered statistically significant when p values were < 0.05.

## Results and discussion

In this study, potential toxic effects of AFB_2_ on albino mice were investigated by using body weight and organ weight, serum parameters, oxidant/antioxidant dynamics and cytogenetic parameters in albino mice and the protective effects of resveratrol against AFB_2_ toxicity were studied. In addition, radical scavenging activity of resveratrol were investigated and associated with its protective effect. Resveratrol exhibits antioxidant activity by neutralizing the free radicals. Free radical scavenging is one of the important mechanism of antioxidant activity. In this study, free radical scavenging activity of resveratrol was tested against superoxide, DPPH and H_2_O_2_, which have important radical properties.

### Radical scavenging activity of resveratrol

**Figure 1:**
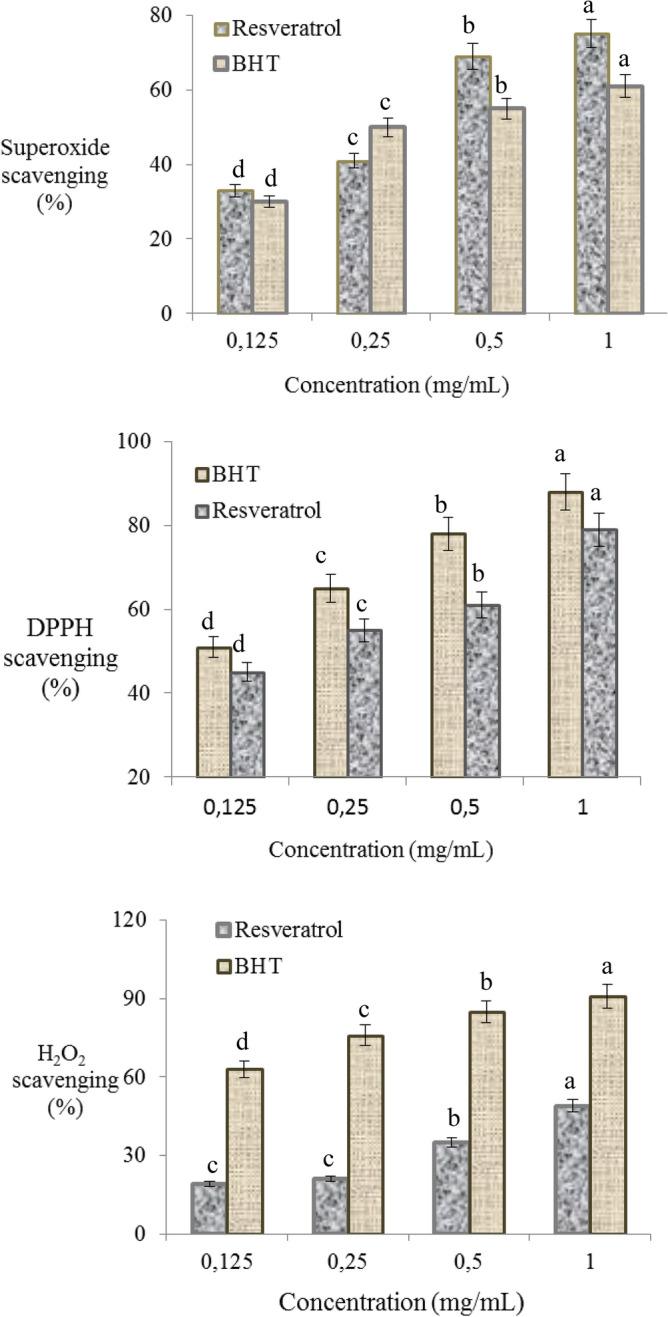
Superoxide, DPPH and H_2_O_2_ scavenging property of resveratrol. The experiment was repeated three times at each concentration. Each histogram is a decimal average. The vertical lines on the bars indicate the standard error. Different letters^(a–d)^ indicated averages p < 0.05 is important.

There is a close relationship between the protective role of resveratrol and its antioxidant activity. Radical scavenging activity plays an important role in antioxidant activity mechanism. The radical scavenging property of resveratrol tested against superoxide, DPPH and H_2_O_2_ and the results are presented in Fig. 1. The scavenging effect of resveratrol also increased with the increasing dose and the highest scavenging effect was observed against DPPH (%79). DPPH, a stable free radical, accepts an electron or hydrogen to become a stable diamagnetic molecule, and 1 mg/mL resveratrol and BHT exhibited 79% and 88% DPPH scavenging activity, respectively. Among free radicals, superoxide is a strong oxidizing agent, and resveratrol exhibited a stronger superoxide scavenging activity than the standard antioxidant compound. Especially at high doses (0.5–1 mg/mL), resveratrol exhibited higher superoxide scavenging activity compared to the BHT. H_2_O_2_ itself is not very reactive, however, it can sometimes be toxic to cells as it mediates the formation of the hydroxyl radical^22^. Resveratrol showed a moderate hydrogen peroxide scavenging activity and the peroxide scavenging activity of 1 mg/mL resveratrol was 49%. Resveratrol has electron donor properties to neutralize free radicals and provides the formation of stable products with its reducing properties. Its reducing property prevents the initiation of radical chain reactions and protects cells against oxidation^22^.

Literature studies show that resveratrol is a good radical scavenging agent^20,21^. Gülçin^22^ reported that resveratrol has a higher superoxide scavenging activity compared to standard antioxidants such as α-tocopherol and trolox. Murcia and Matinez-Tome^36^ indicated that resveratrol tested at different concentrations did not exhibit H_2_O_2_ removal activity, whereas Gülçin^22^ reported that 30 μg/mL resveratrol had 19.5% H_2_O_2_ scavenging activity. These results indicate that resveratrol is a powerful antioxidant and will have protective properties against AFB_2_ toxicity.

### Physiological parameters

The effects of AFB_2_ and resveratrol applications on body weight and organ weights are given in Table 2. In control group, the initial body weight average was 33.50 ± 1.80 g, and a weight increase of approximately 12.40 g was observed at the end of the application period. Similar increase was observed in Groups II and III where 10 µg/kg and 20 µg/kg b.w resveratrol were administered, respectively. This result shows that resveratrol application alone does not cause any toxic effect on weight gain in organisms. The most significant reduction in body weight was observed in the group treated with 20 µg/kg b.w AFB_2_, and a 23% reduction observed compared to the control group. Abnormalities caused by AFB_2_ application on body weight were also observed in liver and kidney weights. While no statistically significant difference observed in the organ weights of groups I, II and III (p > 0.05), statistically significant reductions were found in 20 µg/kg b.w AFB_2_ treated group (p < 0.05). Liver and kidney weights decreased by 46.9% and 46.3% times, respectively, in group IV compared to the control group. This result indicates that liver and kidney weights were similarly affected by AFB_2_ toxicity. The negative effects of AFB_2_ treatment on body weight and organ weights in albino mice may be associated with anorexia, fatigue, impaired protein/lipid metabolism, protein synthesis and inhibition of lipogenesis. Lipolysis-lipogenesis balance plays an important role in increasing body weight and weight gain is increased by lipogenesis and fat accumulation^37,38^. Lipid metabolism disorder, which may arise as a result of the hepatotoxic effects of aflatoxins and their negative effects on other tissues, may significantly affect the weight gain and organ weights in organisms. Although there is no study in the literature about the effect of AFB_2_ application on weight gain, there are important data on the effects of other aflatoxin species. Similarly to our results, Arvind and Churchil^39^ found that there was a 33.94% reduction in weight gain of chickens fed with 1 ppm AFB_1_ for 6 weeks. In another study, Dimitri et al.^40^ reported that exposure to AFB_1_ and AFG_2_ caused an average of 539 g (16%) body weight loss in rabbits, and this was related to impaired protein synthesis and DNA synthesis. Cardoso et al.^41^ stated that the mixture containing 62.9% of AFB_1_ and 37.1% of AFG_1_ significantly decreased the body weight gain in broiler chicks, did not cause a change in liver anatomy in terms of shape, texture and color, however, it caused an increase in relative liver weight.

**Table 2:** Effects of AFB_2_ and resveratrol application on body and organ weights.

Groups	Initial body weight (g)	Final body weight (g)	Liver weight (g)	Kidney weight (g)
Group I	33.50 ± 0.46^a^	45.90 ± 0.55^a^	2.30 ± 0.09^a^	1.60 ± 0.08^a^
Group II	33.76 ± 0.50^a^	45.72 ± 0.53^a^	2.24 ± 0.08^a^	1.57 ± 0.07^a^
Group III	33.34 ± 0.43^a^	44.96 ± 0.51^a^	2.19 ± 0.08^a^	1.52 ± 0.06^a^
Group IV	33.58 ± 0.48^a^	35.36 ± 0.46^d^	1.22 ± 0.02^d^	0.86 ± 0.03^d^
Group V	33.89 ± 0.48^a^	37.90 ± 0.48^c^	1.50 ± 0.03^c^	1.10 ± 0.04^c^
Group VI	33.66 ± 0.46^a^	41.16 ± 0.50^b^	1.78 ± 0.05^b^	1.34 ± 0.05^b^

Values are shown as mean ± SEM (n = 6). Group I: control, Group II: 10 mg/kg b.w resveratrol, Group III: 20 mg/kg b.w resveratrol, Group IV: 20 µg/kg b.w AFB_2_, Group V: 10 mg/kg b.w resveratrol + 20 µg/kg b.w AFB_2_, Group VI: 20 mg/kg b.w resveratrol + 20 µg/kg b.w AFB_2_. Data shown with different letters^(a–d)^ in the same column are statistically significant (p < 0.05).

Another important result observed in the body weight and organ weight analysis is that resveratrol application together with AFB_2_ caused weight gain and an increase in organ weights. An increase observed in the body weights of Groups V and VI, in which resveratrol and AFB_2_ were applied together, compared to the group treated with 20 µg/kg b.w AFB_2_. This improvement was more pronounced in Group VI, which received 20 mg/kg resveratrol. While an increase of 1.78 g in body weight observed only in the group receiving AFB_2_, this increase determined as 7.5 g in the group in which AFB_2_ and 20 mg/kg resveratrol were administered together. The increasing effect of resveratrol on body weight was also observed in kidney and liver organ weights. In Group VI, where 20 mg/kg b.w resveratrol was applied, liver and kidney weights increased by 45.9% and 55.8%, respectively, compared to the group treated with AFB_2_. This healing property of resveratrol can be explained by the suppression of toxic effects induced by AFB_2_. Resveratrol exhibits an important protective role in lipid metabolism by preventing lipid peroxidation and LDL oxidation, decreasing total cholesterol and low-density lipoprotein cholesterol levels in plasma, and increasing plasma high-density lipoprotein (HDL) levels^17,42^. This protective role reduced the negative effects of AFB_2_ on lipid metabolism and caused a re-increase in body weight. Resveratrol exhibits an important protective role in tissues such as kidney and liver, particularly by reducing oxidative damage and inflammation^43^. The fact that the decrease observed in the weight of kidney and liver tissues as a result of AFB_2_ administration increased again with resveratrol can be explained by this protective feature.

### Liver and kidney functions analysis

**Figure 2:**
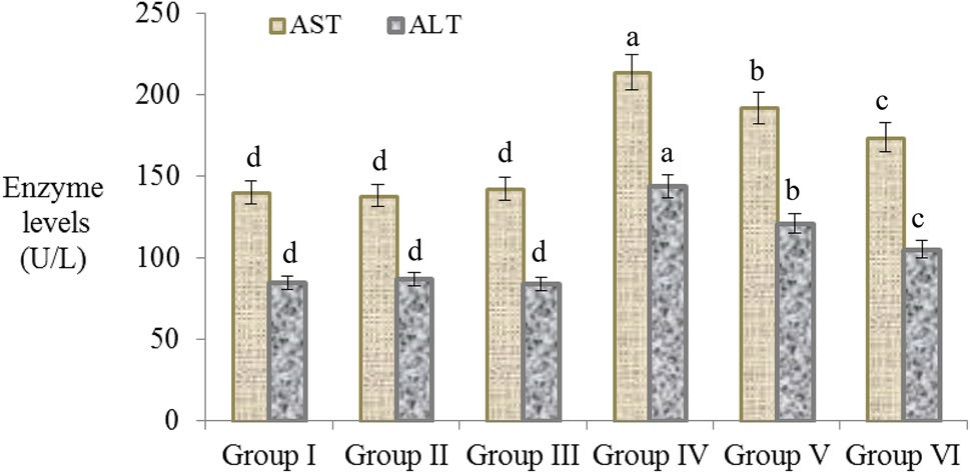
The effects of AFB2 and resveratrol on the levels of AST and ALT, indicator enzymes of liver injury. Group I: control, Group II: 10 mg/kg b.w resveratrol, Group III: 20 mg/kg b.w resveratrol, Group IV: 20 µg/kg b.w AFB_2_, Group V: 10 mg/kg b.w resveratrol + 20 µg/kg b.w AFB_2_, Group VI: 20 mg/kg b.w resveratrol + 20 µg/kg b.w AFB_2_. Values are shown as mean ± SD (n = 6). Each histogram is a decimal average. The vertical lines on the bars indicate the standard error. Different letters^(a–d)^ indicated averages p < 0.05 is important.

The effects of AFB_2_ and resveratrol application on selected serum parameters are given in Fig. 2 and Table 3. Among the serum parameters, the liver marker enzymes AST and ALT, and the kidney damage indicators BUN and creatinine levels were examined. No abnormality was found in AST and ALT levels in groups I, II and III, and the differences between the groups were statistically insignificant (p > 0.05). Significant increase was found in AST and ALT levels in the 20 µg/kg b.w AFB_2_ treated group. AST and ALT activities increased by 52.8% and 69.4%, respectively, in the AFB_2_-treated group compared to the control. These statistically significant increases in AST and ALT levels indicate that AFB_2_ administration causes serious liver damage. Besides carcinogenic and mutagenic effects of aflatoxins, hepatotoxic and nephrotoxic effects are also known. Aflatoxins inhibit polymerase activity in hepatocytes, cause biochemical and histopathological changes in liver and cause hepatocellular necrosis^44^. All these effects induced by aflatoxin cause damage to liver, resulting in cell destruction and organ failure. These abnormalities observed in hepatocytes cause the enzymes in these cells to enter the blood and increase their levels in the serum. AST is found in a variety of tissues, including liver, brain, pancreas, heart, kidney, lung and skeletal muscle. After damage to these tissues, AST passes from the tissue cells to the bloodstream and the blood level increases. Increased AST levels are indicative of tissue damage, but do not indicate liver damage alone. ALT is predominantly found in the liver, and any increase in the blood level of ALT is accepted as a direct indicator of liver damage^45^. The increase in both enzymes in the AFB_2_ applied group is evidence of liver damage. Similarly, Eraslan et al.^46^ reported that a mixture of 500 μg/kg aflatoxin (80% AFB_1_, 10% AFB_2_, 6% AFG_1_, 4% AFG_2_) exposure significantly increased serum ALT, AST, ALP and LDH enzyme activities and these increases were associated with liver damage. Han et al.^47^ reported that 20 μg/kg and 40 μg/kg AFB_1_ administration caused a significant increase in AST and ALT enzymes, and this increase caused by the transition of enzymes from hepatocytes to the blood as a result of liver damage.

**Table 3:** Effects of AFB_2_ and resveratrol on BUN and creatinine levels.

Groups	BUN (mg/L)	Creatinine (mg/L)
Group I	280 ± 2.28^d^	6.15 ± 0.47^d^
Group II	282 ± 2.30^d^	6.10 ± 0.42^d^
Group III	277 ± 2.23^d^	6.12 ± 0.44^d^
Group IV	375 ± 2.98^a^	14.38 ± 0.98^a^
Group V	342 ± 2.74^b^	12.25 ± 0.86^b^
Group VI	308 ± 2.65^c^	9.56 ± 0.78^c^

Values are shown as mean ± SEM (n = 6). Group I: control, Group II: 10 mg/kg b.w resveratrol, Group III: 20 mg/kg b.w resveratrol, Group IV: 20 µg/kg b.w AFB_2_, Group V: 10 mg/kg b.w resveratrol + 20 µg/kg b.w AFB_2_, Group VI: 20 mg/kg b.w resveratrol + 20 µg/kg b.w AFB_2_. Data shown with different letters^(a–d)^ in the same column are statistically significant (p < 0.05).

AFB_2_ administration also caused an increase in BUN and creatinine levels, which are indicators of kidney damage. BUN and creatinine levels increased by 33.9% and 134.4%, respectively, in 20 µg/kg b.w AFB_2_ treated group compared to the control group. Aflatoxins cause different damages in kidney such as glomerular capillary damage, obstruction in cortical blood vessels, coagulation necrosis, inflammation, focal bleeding^48^. BUN and creatinine levels are used to predict potential kidney damages. Creatinine is the breakdown product of creatinine phosphate in muscle and is usually produced by the body at a constant rate depending on muscle mass and excreted through the kidneys. Since creatinine cannot be filtered sufficiently in kidney damage or failure, its level increases in the blood. Similarly, BUN levels increase in kidney disease or failure, fever and shock, obstruction of the urinary tract due to kidney stones^49^. In this study, administration of 20 µg/kg AFB_2_ caused significant increases in BUN and creatinine levels, indicating kidney damage. Supporting these findings, Eraslan et al.^46^ reported that 500 μg/kg aflatoxin exposure resulted in significant increases in uric acid, BUN and creatinine levels in albino rats, indicating kidney damage. Verma and Kolhe^50^ reported that 15 mg/kg aflatoxin isolated from *Aspergillus parasiticus* significantly increased the serum creatinine level in rabbits and caused nephrotoxicity.

While AFB_2_ administration alone caused a significant increase in liver and kidney indicator parameters, resveratrol application together with AFB_2_ improved alterations in these parameters. ALT and AST levels decreased by 27.1% and 18.7%, respectively, in Group VI, compared to the group that only applied AFB_2_. Despite this decrease, it was determined that AST and ALT levels were still higher than the control group. When the kidney marker parameters were examined, BUN and creatinine levels were decreased by 17.9% and 33.5%, respectively, compared to the AFB_2_ applied group. The decline in increased ALT, AST, BUN and creatinine levels after resveratrol application can be explained by the protective properties of resveratrol. Resveratrol exhibits an important protective role in kidney and liver, especially by reducing macromolecular oxidation, lipid peroxidation and inflammation^43^. Although there is no study on the protective effect of resveratrol against AFB_2_-induced damages in the literature, it has been reported that resveratrol reduces liver and kidney damages induced by many chemicals. Şener et al.^51^ reported that the administration of 100 mg/kg of resveratrol treatment exhibited an antioxidant protection and prevented liver damage by reducing induced oxidative damage in rats. Palsamy and Subramanian^52^ found that 5 mg/kg resveratrol resulted in an improvement in creatinine clearance and had protective properties on the kidney by reducing oxidative stress and inflammation.

### Antioxidant/oxidant dynamic

**Figure 3:**
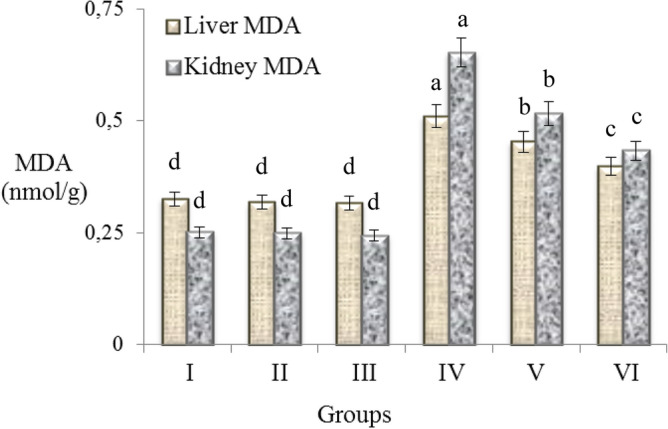
Effects of AFB_2_ and resveratrol on MDA levels, an indicator of lipid peroxidation in liver and kidney cells. Group I: control, Group II: 10 mg/kg b.w resveratrol, Group III: 20 mg/kg b.w resveratrol, Group IV: 20 µg/kg b.w AFB_2_, Group V: 10 mg/kg b.w resveratrol + 20 µg/kg b.w AFB_2_, Group VI: 20 mg/kg b.w resveratrol + 20 µg/kg b.w AFB_2_. Each histogram is a decimal average. The vertical lines on the bars indicate the standard error. Different letters^(a–d)^ indicated averages p < 0.05 is important.

**Figure 4:**
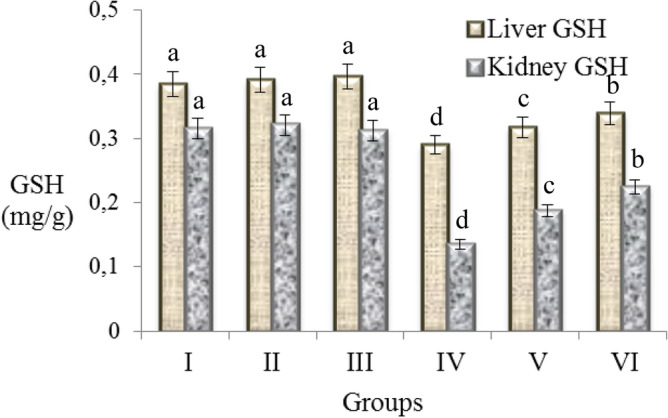
Effects of AFB_2_ and resveratrol on the levels of GSH, a potent endogenous antioxidant, in liver and kidney cells. Group I: control, Group II: 10 mg/kg b.w resveratrol, Group III: 20 mg/kg b.w resveratrol, Group IV: 20 µg/kg b.w AFB_2_, Group V: 10 mg/kg b.w resveratrol + 20 µg/kg b.w AFB_2_, Group VI: 20 mg/kg b.w resveratrol + 20 µg/kg b.w AFB_2_. Each histogram is a decimal average. The vertical lines on the bars indicate the standard error. Different letters^(a–d)^ indicated averages p < 0.05 is important.

Similar to the negative effects of AFB_2_ administration on physiological and serum parameters, a toxic effect was also observed on antioxidant/oxidant dynamic. The effects of exogenous and endogenous oxidant molecules in the body neutralized by antioxidants. The increase in exogenous oxidant molecules and an insufficiency of endogenous antioxidants cause deterioration in antioxidant/oxidant balance. In order to examine the effects of AFB_2_ on this balance, MDA and GSH levels in liver and kidney were examined and the results are given in Figs. 3 and 4. It was observed that MDA levels were close to each other in the control group and only resveratrol treated groups. 20 µg/kg b.w AFB_2_ administration caused a significant increase in MDA levels of kidney and liver. When compared with the control group, AFB_2_ exposure significantly increased the MDA levels in liver and kidney compared to control. Many studies have demonstrated that aflatoxin species cause oxidative stress^9,53,54^. Oxidative stress causes damage and lipid peroxidation in cells. The cellular structure most affected by lipid peroxidation is the cell membrane, which contains saturated and unsaturated lipids. MDA is a mutagenic product formed as a result of peroxidation of membrane lipids^55,56^. At the end of the normal biochemical process, low levels of MDA can occur in cells and can be removed with the antioxidant system.

Increased MDA level in AFB_2_ applied group indicates that AFB_2_ cause oxidative damage and lipid peroxidation in liver and kidney. Similarly, Shen et al.^57^ reported that cell damage and lipid peroxidation occurred in the liver of rats exposed to AFB_1_. In a similar study, it was reported that the serum MDA level increased in albino rats treated with aflatoxin mixture, and this increase was associated with the formation of free radicals and the weakening of cellular antioxidant defense mechanisms^46^. In this study, liver MDA levels decreased by 11.2% and 21.9% in the groups V and VI treated with 10 mg/kg b.w and 20 mg/kg b.w resveratrol, respectively, compared to the group that received only AFB_2_. In Group V and VI, the decrease in kidney MDA levels was 20.9% and 33.7%, respectively. The most significant improvement in liver and kidney MDA levels observed in the 20 mg/kg b.w resveratrol group (Group VI), but MDA levels in this group were still higher than in the control group. The decreasing effect observed in MDA level can be explained by the antioxidant role of resveratrol. Resveratrol exhibits a protective effect by directly suppressing oxidant molecules and radicals, increasing the expression and activity of antioxidant enzymes such as glutathione peroxidase, superoxide dismutase and catalase^15,16^. Resveratrol reduces lipid peroxidation and can also prevent deterioration in cell membrane integrity^17^. The decrease in MDA levels is closely related to the radical scavenging activity of resveratrol. The ability of resveratrol to scavenge free radicals was determined by previous analyzes in this study. Resveratrol exhibited a significant scavenging effect against superoxide and H_2_O_2_ that cause oxidation in the cell and especially in cell membrane lipids. Supporting our results, Kasdallah-Grissa et al.^58^ reported that increased MDA levels as a result of induced lipid peroxidation in liver, heart, brain and testicular tissues decreased by 72.7–40.5% with resveratrol administration.

The effects of resveratrol and AFB_2_ application on GSH levels are given in Fig. 4. It was observed that GSH levels were close to each other in the control group and only resveratrol treated groups. Resveratrol application was increased the GSH level at a low level in Group II and III, but this increase was statistically insignificant (p > 0.05). 20 µg/kg b.w AFB_2_ administration caused a decrease in GSH levels in liver and kidney. Compared with the control group, AFB_2_ treatment reduced GSH levels in liver and kidney tissues by 24.6% and 57.1%, respectively. Considering the increasing effect of AFB_2_ on MDA levels of liver and kidney and its decreasing effect on GSH levels, it can be said that the oxidant/antioxidant dynamic was disrupted. The increase in MDA level is evidence of oxidative damage, and antioxidant molecules play a role to neutralize this damage. GSH is a non-enzymatic tri-peptide antioxidant and is an essential part of the antioxidant defense in free radical removal. The reduced-GSH protects the cell against oxidative damage by neutralizing free radicals. As a result of this reaction, GSH molecules turn into the oxidized GS-SG state and the level of reduced-GSH decreases^59^. In short, the increase in the MDA ratio and the decrease in reduced-GSH prove the oxidative damage caused by AFB_2_. GSH conjugation also plays an important role in aflatoxin metabolism, and conjugation contributes to the decrease in the level of reduced-GSH^8^. In the literature, it is reported that aflatoxin exposure causes changes in the levels of antioxidant molecules. Kheir Eldin et al.^60^ reported that 250 μg/kg AFB_1_ treatment in rats decreased GSH level and increased lipid peroxidation. Eraslan et al.^46^ found that aflatoxin exposure in rats caused a significant decrease in antioxidant enzyme levels in the liver, thereby disrupting the antioxidant balance. In this study, it was determined that resveratrol application provided improvement in impaired reduced-GSH levels and also with increasing the resveratrol dose, recovery is more predominant. 20 mg/kg b.w resveratrol application provided 11.9% and 28.8% improvement in liver and kidney GSH levels, respectively, compared to the AFB_2_-treated group. Resveratrol scavenges the oxidative stress and free radicals induced by AFB_2_ and prevents the decrease in GSH level. Similarly, Şener et al.^51^ reported that 30 mg/kg of resveratrol administration in mice regulates the impaired antioxidant balance and leads to improvement in decreased GSH levels.

### Genotoxic effects

The genotoxic effects of AFB_2_ and the protective effect of resveratrol against these effects were evaluated by examining MN formation in buccal mucosa epithelium, erythrocyte and leukocyte cells and CAs frequency in bone marrow cells.

### MN assay

**Figure 5:**
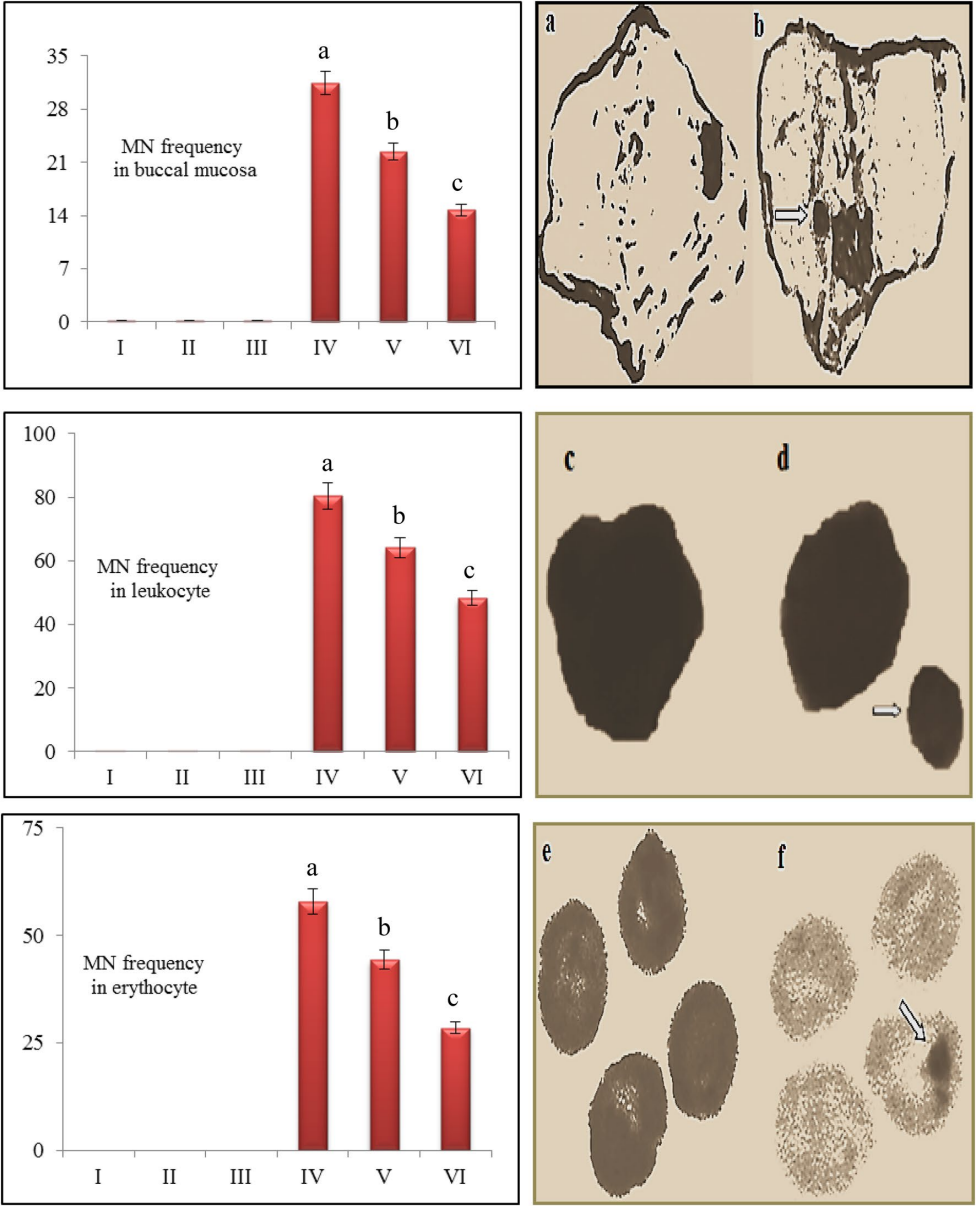
Frequencies and the appearance of MN in buccal mucosa epithelium, erythrocyte and leukocyte cells. Group I: control, Group II: 10 mg/kg b.w resveratrol, Group III: 20 mg/kg b.w resveratrol, Group IV: 20 µg/kg b.w AFB_2_, Group V: 10 mg/kg b.w resveratrol + 20 µg/kg b.w AFB_2_, Group VI: 20 mg/kg b.w resveratrol + 20 µg/kg b.w AFB_2_. (**a**) normal nucleus in the buccal mucosa epithelium cell, (**b**) MN in the buccal mucosa cell, (**c**) normal nucleus in leukocyte cell, (**d**) MN in the leukocyte, (**e**) normal nucleus in erythrocyte cell, (**f**) MN formation in the erythrocyte. Each histogram is a decimal average. The vertical lines on the bars indicate the standard error. Different letters^(a–d)^ indicated averages p < 0.05 is important.

Frequencies and the appearance of MN are presented in Fig. 5. A statistically significant level of MN formation was not observed in the control group and groups II and III, where only resveratrol was applied. These results show that resveratrol application alone does not induce MN formation. High levels of statistically significant MN frequencies were obtained in the group treated with 20 µg/kg b.w AFB_2_ (p < 0.05). Among the three cells tested, the highest MN formation observed in leukocyte cells. MN frequencies ​​observed in buccal mucosa epithelium, erythrocyte and leukocyte cells in the group treated with 20 µg/kg b.w AFB_2_ were 31.46 ± 3.28, 57.92 ± 5.85 and 80.35 ± 7.70, respectively. MN formation caused by chromosomal fragments, double-stranded DNA breaks, single-strand breaks, or lagging chromosomes. In the telophase stage of mitosis, a nuclear envelope formed around the delayed chromosomes and fragments, resulting in smaller MN structures resembling the main nucleus. The increase in MN formation and frequency indicates the presence of toxic effects on the cell. Therefore, MN test is used to evaluate the toxicity of chemicals to which cells are exposed^61,62^. The size of MN formed in a cell provides information about the mechanism of toxicity and also enables the determination of the aneugenic or clastogenic effect of the chemicals. MNs resulting from centromere division errors and spindle defects as a result of the aneugenic effect are larger in size. MN’s formed as a result of clastogenic effect have smaller dimensions since they originate from chromosome fragments or breaks^61,63^. MN formation in buccal mucosa, erythrocyte and leukocyte cells belonging to groups treated with AFB_2_ indicates that AFB_2_ has a genotoxic effect. The observation of MN formations in both large and small sizes indicates that both aneugenic and clastogenic effects of AFB_2_. This result shows that AFB_2_ can induce MN formation by causing centromere division errors, spindle defects or chromosome breaks. Similarly, Ezekiel et al.^53^ reported that the frequency of MN in erythrocytes increased in mice treated with AFB_1_ and that AFB_1_ caused mutagenic and genotoxic effects. Corcuera et al.^64^ observed a toxic effect occurred on bone marrow cells and f MN frequency increased in rats treated with AFB_1_.

In order to determine the genotoxic effects of AFB_2_, CAs frequency in the bone marrow were examined and the results were given in Table 4. In Group I, II and III, no CAs were found except for low level of gap formation (p > 0.05). Similar MN levels of the control group, Groups II and III show that resveratrol did not cause genotoxic effects at doses of 10 mg/kg body weight and 20 mg/kg body weight. High level and statistically significant CAs frequency were detected in the group treated with 20 µg/kg b.w AFB_2_ (p < 0.05). Among these abnormalities, fragment occurred at the highest rate as a level of 53.18 ± 5.50. When all abnormalities are evaluated together, it is possible to sort the CAs according to the frequency of occurrence as break > fragment > acentric chromosome > dicentric chromosome > gap > ring. Observation of these abnormalities proves the genotoxic effect of AFB_2_. The genotoxic effects of aflatoxins are closely related to oxidative stress and damage in living systems. Aflatoxins are metabolized to aflatoxin-8,9-epoxide, which is a reactive form that binds to DNA and forms aflatoxin-N7-guanine structures by binding with high affinity to guanine bases in DNA. Aflatoxin-N7-guanine generates guanine-thymine transversion mutations in DNA and negatively effects the p53 repressor gene in the cell cycle. Bone marrow cells are susceptible to oxidative damage and clastogenic chemicals, so they are widely used for mutagenicity and/or antimutagenicity screening^65,66^. High level of breaks and fragments observed in bone marrow cells indicates the clastogenic effect of AFB_2_. Chromosome and chromatid breaks resulting from clastogenic effect also cause the formation of fragments^67,68^. The high MN level observed in the AFB_2_ applied group was also closely related to the formation of breaks and fragments. In this study, the high level of MN frequency and fragments observed as a result of AFB_2_ application also support each other. Ring chromosomes arise when a chromosome is broken twice and the broken ends are re-united. In mitosis, if the ring chromosomes of some cells cannot be attracted to the poles, chromosome loss/excess may occur in the cells and this can cause genome instability. Gap formations are defined as achromatic lesions that do not exceed the width of a chromatid. Dicentric and acentric chromosomes are also formed as a result of breakage, and the dicentric chromosome mostly occurs as a result of the rearrangement of chromosome breaks. Increased frequency of gaps and dicentric chromosomes are considered important indicators of toxicity^68,69^. When all these effects are evaluated together, it can be said that most of the CAs originate from fragments or breaks. In this study, AFB_2_ caused the most breaks and other abnormalities were observed as a result of the rearrangement of these breaks. There are many studies in the literature reporting the genotoxic effect of other aflatoxin species, especially AFB_1_. Fetaih et al.^68^ reported that AFB_1_ induced the macro-DNA damages such as gap, fracture, deletion, dicentric chromosome, stickiness, hypopolyploidy, centromeric rearrangements in the bone marrow cell of rats. Fadl-Allah et al.^70^ observed that AFB_1_ (5–25 μg/mL) exposure increased the frequency of CAs such as stickiness, bridges, laggards, unequal division, fragments.

**Table 4:** CAs frequency in AFB_2_ and resveratrol treated groups.

CAs type	Group I	Group II	Group III	Group IV	Group V	Group VI
Break	N.O	N.O	N.O	53.18 ± 1.42^a^	40.10 ± 1.38^b^	26.94 ± 1.16^c^
Fragment	N.O	N.O	N.O	38.76 ± 1.24^a^	26.37 ± 1.14^b^	17.48 ± 0.98^c^
Acentric	N.O	N.O	N.O	25.14 ± 1.10^a^	16.53 ± 1.10^b^	11.40 ± 0.90^c^
Dicentric	N.O	N.O	N.O	18.74 ± 1.00^a^	11.63 ± 0.90^b^	7.35 ± 0.52^c^
Gap	0.22 ± 0.13^d^	0.14 ± 0.08^d^	0.11 ± 0.06^d^	13.78 ± 0.96^a^	8.66 ± 0.86^b^	3.75 ± 0.55^c^
Ring	N.O	N.O	N.O	9.67 ± 0.88^a^	5.16 ± 0.68^b^	1.88 ± 0.48^c^

Values are shown as mean ± SEM (n = 6). Data shown with different letters^(a–d)^ in the same line are statistically significant (p < 0.05). Group I: control, Group II: 10 mg/kg b.w resveratrol, Group III: 20 mg/kg b.w resveratrol, Group IV: 20 µg/kg b.w AFB_2_, Group V: 10 mg/kg b.w resveratrol + 20 µg/kg b.w AFB_2_, Group VI: 20 mg/kg b.w resveratrol + 20 µg/kg b.w AFB_2_.

*N.O* not observed.

Another important result found in genotoxicity analyzes is that resveratrol application exhibits protective effect against AFB_2_-induced CAs and MN formation. The frequency of CAs and MN formation decreased with the increase of resveratrol dose. Application of resveratrol together with AFB_2_ reduced MN frequency in buccal mucosa epithelium, erythrocyte and leukocyte cells. Similarly, there was a decline in CAs frequency in bone marrow after resveratrol application. The most prominent protective effect of resveratrol was observed against gap formation. 20 mg/kg b.w resveratrol exhibited a reducing effect in the range of 49.34–80.5% in CAs frequency induced by AFB_2_. This healing effect observed against MN and CAs formations can be explained by the multi-biological properties of resveratrol.

### Recovery effects of resveratrol

**Figure 6:**
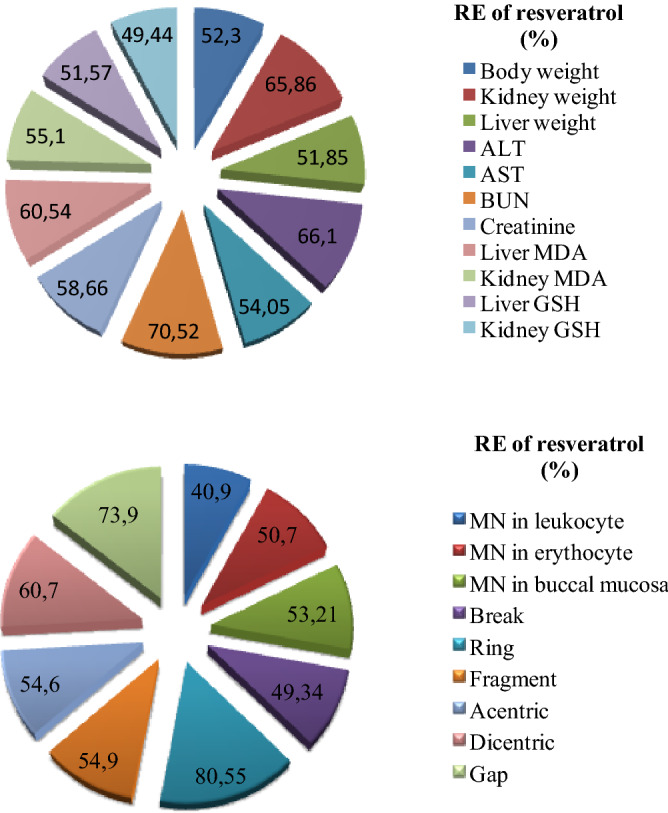
Recovery effects of 20 mg/kg resveratrol against AFB_2_ toxicity on physiological, biochemical and genotoxic parameters.

RE values of 20 mg/kg b.w resveratrol in all tested parameters are given in Fig. 6. Resveratrol, which exhibits a RE value in the range of 40.9–80.55%, exhibited the highest protective role against ring formation. Although RE values against GSH decrease in kidney, MN formation in leukocyte and fragment formation in bone marrow were below 50%, resveratrol exhibited a RE over 50% in all other toxicity parameters. Resveratrol ensured the normalization of antioxidant/oxidant balance and resulted significant improvements in kidney and liver marker parameters. This protection can be explained by the suppression of oxidative damage induced by AFB_2_. The protective role of resveratrol on the kidney and liver was proven by causing an improvement in organ weights, a decrease in organ MDA and an increase in GSH. In the literature, resveratrol has been reported to have a protective role in the liver by alleviating oxidative stress, induced apoptosis, necrosis and inflammation^71^. It is also reported that resveratrol administration reduces oxidative damage in kidney by enhancing the antioxidant defense^72^. Resveratrol also exhibited a significant RE against the formation of MN and CAs and the highest RE values were observed against gap and ring formations. The protective role of resveratrol against cytogenetic toxicity can be explained by its strengthening effect on antioxidant system and its regulatory role on the cell cycle. Resveratrol has a powerful antioxidant activity that scavenges free radicals and induces antioxidants such as GSH and CAT^20,21^. The strong radical scavenging properties of resveratrol have been demonstrated by different analyzes in this study. Its protective role against CAs and MN damages is also due to this scavenging effect. Resveratrol also has a regulatory role in the cell cycle. It enables the cell to remain in the G_0_/G_1_ phase or S phase by suppressing the cell cycle in damaged cells. It may also regulate the cell cycle by inhibiting the expression of cyclin D_1_, D_2_, E and decreasing the expression of cyclin-dependent kinases^73,74^. In this way, it can reduce the frequency of DNA damage and CAs by preventing damaged cells from dividing. Similar studies in the literature also support our results. Macar et al.^75^ reported the attenuating effect of resveratrol administration against CAs such as fragment, sticky chromosome, unequal chromatin distribution, bridges and abnormal polarization.

## Conclusion

In this study, toxic effects of AFB_2_ on albino mice and the protective effects of resveratrol against these toxic effects were investigated. Significant reductions in body weight, kidney and liver weights were detected in mice treated with AFB_2_. Exposure to AFB_2_ caused deterioration of antioxidant/oxidant balance in kidney and liver and a significant increase in indicator parameters of these organs. The induction of MN and CAs formation also reveals the genotoxic effects of AFB_2_. Another important finding obtained from the study is determination of the protective effect of resveratrol against the physiological, biochemical and genotoxic damages of AFB_2_. Protective effects of resveratrol increased depending on the dose, but this increase was not directly proportional. The protective property of resveratrol associated with its superoxide, H_2_O_2_ and DPPH radical scavenging activity and its antioxidant role.

Although there are many studies conducted with aflatoxin species in the literature, there is no study investigating the multiple toxic effects of AFB_2_. Especially, with this study the first data entry was provided to the literature regarding that AFB_2_ induces MN formation in the buccal mucosa epithelium. There are many data in the literature regarding the protective properties of resveratrol, which has antioxidant properties. However, there is no study reporting the protective properties of resveratrol against toxicity induced by AFB_2_. In this respect, this study is the first to report that resveratrol exhibits a dose-dependent protective role against AFB_2_ toxicity. In the sustainability of high quality of life, it is very important to study the toxic effects of chemicals contaminated to organisms and to reduce these effects. From this point of view, this study will direct and guide many studies examining the action mechanisms of toxic agents and protective role of natural products against toxicity.
